# Controlled Payload Release by Magnetic Field Triggered Neural Stem Cell Destruction for Malignant Glioma Treatment

**DOI:** 10.1371/journal.pone.0145129

**Published:** 2016-01-06

**Authors:** Megan E. Muroski, Ramin A. Morshed, Yu Cheng, Tarun Vemulkar, Rhodri Mansell, Yu Han, Lingjiao Zhang, Karen S. Aboody, Russell P. Cowburn, Maciej S. Lesniak

**Affiliations:** 1 Northwestern University Feinberg School of Medicine, 676 N. St Clair Street, Suite 2210, Chicago, IL, 60611, United States of America; 2 The Brain Tumor Center, The University of Chicago, 5841 S. Maryland Avenue, MC 3026, Chicago, IL, 60637, United States of America; 3 Shanghai East Hospital, The Institute for Biomedical Engineering and Nano Science, Tongji University School of Medicine, Shanghai 200029, China; 4 Thin Film Magnetism Group, Cavendish Laboratory, University of Cambridge, JJ Thomson Avenue, Cambridge CB3 0HE, United Kingdom; 5 Department of Neurosciences and Division of Neurosurgery, Beckman Research Institute of City of Hope, Duarte, California, United States of America; University Hospital of Navarra, SPAIN

## Abstract

Stem cells have recently garnered attention as drug and particle carriers to sites of tumors, due to their natural ability to track to the site of interest. Specifically, neural stem cells (NSCs) have demonstrated to be a promising candidate for delivering therapeutics to malignant glioma, a primary brain tumor that is not curable by current treatments, and inevitably fatal. In this article, we demonstrate that NSCs are able to internalize 2 μm magnetic discs (SD), without affecting the health of the cells. The SD can then be remotely triggered in an applied 1 T rotating magnetic field to deliver a payload. Furthermore, we use this NSC-SD delivery system to deliver the SD themselves as a therapeutic agent to mechanically destroy glioma cells. NSCs were incubated with the SD overnight before treatment with a 1T rotating magnetic field to trigger the SD release. The potential timed release effects of the magnetic particles were tested with migration assays, confocal microscopy and immunohistochemistry for apoptosis. After the magnetic field triggered SD release, glioma cells were added and allowed to internalize the particles. Once internalized, another dose of the magnetic field treatment was administered to trigger mechanically induced apoptotic cell death of the glioma cells by the rotating SD. We are able to determine that NSC-SD and magnetic field treatment can achieve over 50% glioma cell death when loaded at 50 SD/cell, making this a promising therapeutic for the treatment of glioma.

## Introduction

Stem cell carriers including neural and mesenchymal stem cells (NSCs and MSCs, respectively) are promising targeted delivery vehicles because of their inherent tumor-tropic migratory behavior. Their ability to improve intratumoral distribution of several cancer therapies has been demonstrated for therapeutic cargoes[1] such as therapeutic proteins[2–4], prodrug-activating enzymes[5, 6], oncolytic viruses[7, 8], and therapeutic nanoparticles.[9–11]

Drug delivery using micro and nanoparticles is of particular interest given the potential therapeutic flexibility of these particles, including material composition, geometric structure, and appendable ligand molecules. The partnership between stem cell carriers and nanoparticles has been applied to several different cancer types *in vivo* including malignant glioma[9, 12, 13], hepatocellular carcinoma[14], breast cancer[15], and lung adenocarcinoma.[16] An initial example of this partnership was the use of iron oxide magnetic nanoparticles to label stem cells for tracking by magnetic resonance imaging.[17, 18] More recently, both NSCs and MSCs have enhanced the distribution of particles for therapeutic purposes.[19] In the case of drug-delivery, lipid nanocapsules, polymeric nanoparticles, gold nanoparticles, and mesoporous silica nanoparticles conjugated with chemotherapy agents (e.g. doxorubicin and coumarin-6) have been loaded intracellularly or onto the surface of stem cell carriers, allowing for delivery of these agents to distant tumor sites.[9–11] Delivery of NSCs carrying doxorubicin-loaded mesoporous silica nanoparticles demonstrated significantly improved survival in a preclinical *in vivo* model of orthotopic glioblastoma in which stem cells were administered into the cerebral hemisphere contralateral to the site of the tumor.[9] NSCs have also been used to improve gold nanorod-mediated photothermal therapy in a subcutaneous tumor model of triple-negative breast cancer, leading to decreased tumor recurrence.[15]

However, many obstacles still limit the efficacy of these cell-based carrier platforms. One limitation for stem cell delivery of drug-conjugated nanoparticles is the potential inefficiency of drug release. While stem cells may be able to release drug-loaded nanoparticles to some extent as they undergo cell death, a certain quantity of this therapeutic cargo may be “consumed” by the carriers themselves either by metabolism of active drug molecules or linking of nanoparticles to cellular components preventing release. Another limitation to such a method is the inability to remotely trigger the timed release of the therapeutic cargo. While photothermal therapy in response to an externally applied near-infrared laser may overcome this hurdle in subcutaneous tumor models, this method may be difficult for inaccessible malignant gliomas.

One method for cellular destruction that may overcome these obstacles is to mechanically disrupt the cell membrane with magnetic nanoparticles controlled by the application of a magnetic field (MF).[20] A number of reports have demonstrated this approach in the destruction of cancer cells.[20–22] For example, spin-vortex magnetic nanodiscs have been used previously to disrupt the membranes of glioma cells *in vitro* upon exposure to a low-frequency alternating MF, eventually triggering cell death in up to 90% of cells.[20] Iron oxide nanoparticles targeting epidermal growth factor receptor (EGFR), upon localization to the cellular lysosome, have been found to induce lysosomal permeabilization, reactive oxygen species production, and cancer cell death upon exposure to an alternating MF.[22] While such results demonstrate a novel means of destroying cancer cells in an efficient manner, these particles still face the obstacle of achieving adequate distribution throughout a tumor bed after local injection. In the context of cell carrier-mediated delivery of nanoparticles, we hypothesized that particle loaded NSCs and the field-induced mechanical destruction mechanism could allow for improved distribution of these therapeutic magnetic nanoparticles as the NSCs are able to release therapeutic particles to neighboring cancer cells.

In this article, we demonstrate that spinning disk (SD) particles can be internalized into NSCs without affecting their ability to migrate. A controlled MF successfully triggers the release of the SD particles, which then allows the particles to be internalized by glioma cells. The immortalized human HB1.F3.CD NSC line displays patho-tropism to the brain and are thus well-suited as a delivery system for targeted delivery of therapeutic agents to glioma. This novel delivery platform has a distinct advantage over current therapeutic systems, as the particles do not have functional surface ligands and therefore are not affected by proteins that may electrostatically associate with the surface. Instead the therapeutic benefit of the particle is dependent on an externally controlled a magnetic field. In addition, in the absence of a MF, we have previously demonstrated that similar particles have a minimal effect on healthy cells.[20]

## Materials and Methods

### Cell culture

U87 human glioma cell line was purchased from the American Type Culture Collection (Manassas, VA, USA). HB1.F3.CD human NSC line originated from the human fetal brain and was modified to constitutively express v-*myc* and cytosine deaminase (CD).[5] The cells were cultured at 37°C with 5% CO_2_ and maintained in Dulbecco's Modification of Eagle's Medium (DMEM) (Mediatech Inc., Manassas, VA, USA), containing 2% penicillin and streptomycin antibiotic (Cellgro, Manassas, VA, USA) and 10% fetal bovine serum (FBS) (Atlanta Biologicals, Lawrenceville, GA, USA). Green fluorescent protein (GFP) and firefly luciferase (Luc) expressing HB1.F3.CD and U87 cell lines were generated according to the literature.[8] To determine uptake of the SD, images of the SD internalized after 24h were analyzed through Image J. Briefly, The cells are washed before imaging to ensure that the particles counted are the particles that are internalized in the cells, over 500 cells were used for analysis. The cells were individually counted in triplicate and the particles were analyzed through binary analysis to measure the specific area as the particles have a negative contrast in the images, the calculated area is then divided by the number of the cells in the image.

### Fabrication of antiparallel magnetic particles

**Fig 1:**
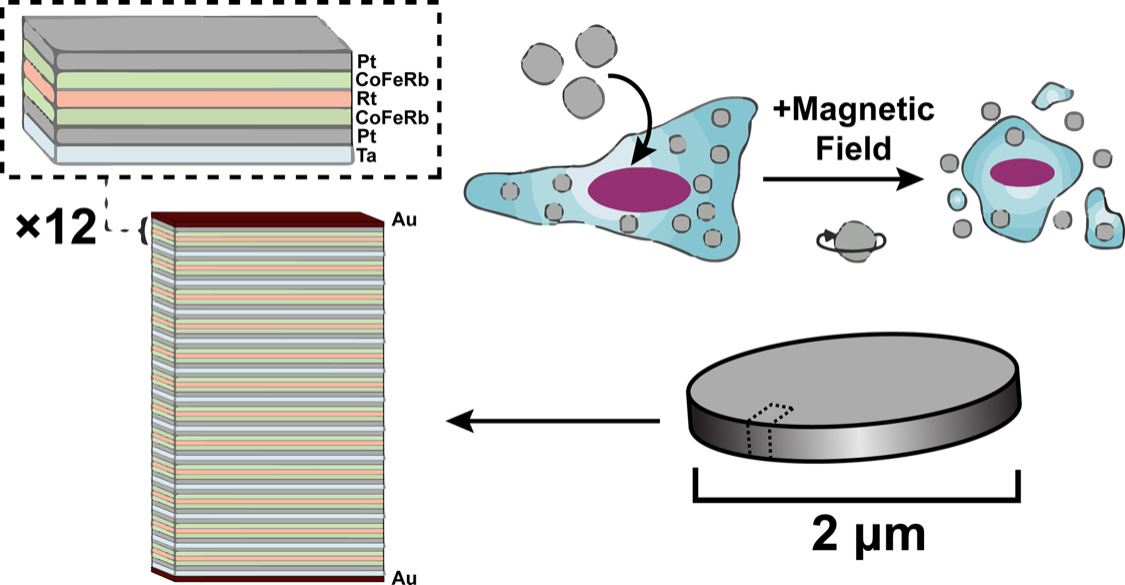
Schematic of the SD particles. The particles are 2 μm x 2 μm with a 60 nm total thickness. A schematic of the magnetic unit is shown at the top. This unit is repeated 12 times. In addition, the particles are capped with an Au layer. The particles are taken into the cell and do not cause death until the particles are exposed to a MF.

The SD particles consist of pairs of 0.9 nm thick CoFeB magnetic layers which are antiferromagnetically coupled via Ruderman-Kittel-Kasuya-Yosida (RKKY) interactions through a Pt (0.4 nm)/Ru (0.9 nm)/Pt (0.4 nm) spacer layer. Each pair of magnetic layers is grown on Ta (2 nm) / Pt (2 nm) buffer with a 2 nm Pt cap. This is repeated 12 times. The repeated layers are capped top and bottom with 5 nm of Au as demonstrated in Fig 1. [23] The total stack is: Au (5 nm) / [Ta (2 nm) / Pt (2 nm) / CoFeB (0.9 nm) / Pt (0.4 nm) / Ru (0.9 nm) / Pt (0.4 nm) / CoFeB (0.9 nm) / Pt (2 nm)]_12_ / Au (5 nm). This magnetic multilayer stack is grown using DC magnetron sputtering onto lithographically patterned pillars of MA-N 1410 photoresist. The photoresist pillars are then dissolved in acetone, releasing the 2 micron wide particles into solution. The particles are then rinsed 3 times in acetone and transferred into water, with three subsequent water rinses. They have a magnetic easy axis which lies perpendicularly to the plane of the particle. At zero field, the antiferromagnetic coupling within each pair causes the layers to point in opposite directions, hence the particles have no net magnetic moment. Applying a field along the easy axis of more than ~1.5–2 kOe saturates the magnetization. The particles have an anisotropy field (the field required to saturate the magnetic moment in the plane of the particle) of 7.5 kOe. Under a rotating MF strong enough to saturate them along their easy axis, the SD particles try to rotate in order to keep their magnetization aligned both with the field and their easy axis. In the process, they apply a torque to their surroundings which can mechanically damage the cells. This is a promising platform for MF controlled drug release.

### Scanning electron microscopy

Following the third rinse in acetone, a few drops of the acetone-MPs suspension were dropcast on to a silicon chip and the acetone was allowed to dry off. The silicon chip was then lightly rinsed with acetone and iso-propyl alcohol and dried under a nitrogen stream. The MPs deposited on the chip were then imaged at a beam voltage of 5 kV in a Philips XL-30 scanning electron microscope.

### Transmission electron microscopy

2.5×10^6^ HB1.F3.CD cells were plated into a T-75 plate for 24h. After the cells were allowed to adhere for 24h, 1.25×10^8^ SD were added into the well and incubated with the cells for 24h. The the cells were then rinsed 3 times in PBS and fixed with 2% glutaraldehyde and 4% paraformaldehyde in 0.1 M sodium cacodylate buffer for 2h. The cell pellet was subsequently washed with sodium cacodylate buffer 3 times and post fixed with 1% osmium tetroxide in 0.1 M sodium cacodylate buffer for 1h. The cells were then washed with sodium cacodylate buffer and maleate buffer, respectively. 1% uranyl acetate in maleate buffer was applied to the cells for 1 hour and then washed 3 times with maleate buffer. Next, the cells were dehydrated, achieved through a series of washes with increased ethanol concentration and introduced to a 2:1 propylene oxide:spurr resin, 1:1 propylene oxide:spurr resin and 100% spurr resin, and the sample was polymerized overnight at 60°C. The resin block was cut using Reichert-Jung Ultracut E microtome into serial 90 nm thick sections and stained with uranyl acetate and lead citrate. Images were taken under 300 kV using a FEI Tecnai F30 microscope.

### Rotating magnet test station

The rotating magnetic field station used an NdFeB Halbach Array magnet (Bunting Magnetics Europe Ltd., Hertfordshire, UK), which produces a uniform 1 Tesla magnetic field diametrically across the central air gap. The magnet was mounted on a motor to control its rotation and the head of the mice was placed inside the central air gap. The test station was used for both in vitro and in vivo experiments.

### Assessment of SD particle toxicity towards HB1.F3.CD cells

HB1.F3.CD cells were seeded into 8-well polystyrene strip plates (5000 cells/well) and incubated with particles at 10, 20, 50 particle per cell ratio. 24h after incubation, cells were treated under rotating MF (1 Tesla, 20Hz) for 30 min. Cell viability was measured using an MTT colorimetric assay 24h post-treatment. 10 μL of MTT labeling reagent 3-[4,5-dimethylthiazol-2-yl]-2,5-diphenyl tetrazolium bromide (Roche Applied Sciences, Indianapolis, USA) was added into the wells and incubated for 4h. 100 μL of 10% SDS solubilization solution was added into the wells to dissolve the purple formazan salt crystals. The plates were read on GeneMate microplate reader. Four replicates were included for each condition. Optical microscopy images of HB1.F3.CD cells loaded with particles were taken before and after treatment using a Nikon Eclipse TS-100 microscope.

### HB1.F3.CD migration assay

1.5x10^4^ HB1.F3.CD cells were seeded into the left and right chambers of a 2-chamber culture-insert (Ibidi, USA). HB1.F3.CD cells were unmodified as a control or further incubated with SD particles at 50 particle/cell ratio. After 24h of incubation, cells were washed 3 times with 10% FBS DMEM. The 2-chamber culture-insert was removed to leave a ~500 μm gap between the two cell lines. Cell migration was monitored over the course of 24h. Briefly, the cells were measured from the movement of the cells in the initial blank rectangle, the experiment was repeated in triplicate. Images were taken using a Nikon Eclipse TS-100 microscope.

### Destruction of glioma cells via SD-loaded HB1.F3.CD cells under MF

For SD loading and release studies, HB1.F3.CD cells were seeded in 96-well plates at a density of 10^4^ cells per well and incubated with SD for 24h (50 SD/cell). HB1.F3.CD cells were then washed 3 times in 10% FBS DMEM, exposed to MF treatment and tested for viability and health 24h later. The number of SD that are exocytosed were expected to be minimal over the course of a few days, as larger particles exocytose at slower rates compared to smaller particles.[24, 25]

For the co-culture experiments, HB1.F3.CD cells were seeded in 96-well plates at a density of 10^4^ cells per well and incubated with SD for 24h (50 SD/cell). Cells were then washed 3 times in 10% FBS DMEM and exposed to MF treatment. MF treatment consists of applying a 1T field rotating at 20 Hz for 30 min. After treatment, further incubated with the green fluorescent protein (GFP) and firefly luciferase (Luc) expressing U87- GFP-Luc cells at a 0.5:1, 1:1, 1:5 ratio (U87-GFP-Luc:HB1.F3.CD). Co-cultured cells received daily MF treatment for 3 days (30 min/day). Cytotoxicity of SD-loaded HB1.F3.CD cells towards U87-GFP-Luc cells was monitored by fluorescence imaging. 96h after co-culture, U87 cell viability was quantified via luciferase assay kit (Promega Corp, Madison, WI) according to the manufacturer's protocol. Briefly, cells were lysed with passive lysis buffer and freeze-thawed by incubating at -80°C for 1h and thawed at room temperature. 5 μL cell lysate from each well was added into 50 μL luciferase assay substrate and bioluminescence was recorded by a GloMax 20/20 Luminometer (Promega Corp, Madison, WI). 4 replicates were included for each condition and repeated twice.

### Confocal microscopy

2x10^4^ HB1.F3.CD cells or U87 cells were seeded on top of microscope glass coverslips. SD particles (10^6^ SD/mL) were added to the cells. After 24 hours incubation, cells were washed 3 times with 10% FBS DMEM. To assess the permeability, cells were incubated with 7-AAD (Biolegend, USA) in DMEM and then received the MF treatment. Images were taken by a Leica SP5 II STED-CW super-resolution laser scanning confocal microscope. To study the SD release, 2x10^4^ SD-loaded HB1.F3.CD cells were co-cultured with U87-GFP-Luc on microscope glass coverslips and treated under MF for 30 min. Confocal images were taken 24 hours post treatment to identify SD particles in glioma cells.

## Results and Discussion

### NSCs are able to efficiently internalize SD particles and are sensitive to a MF trigger for their release

**Fig 2:**
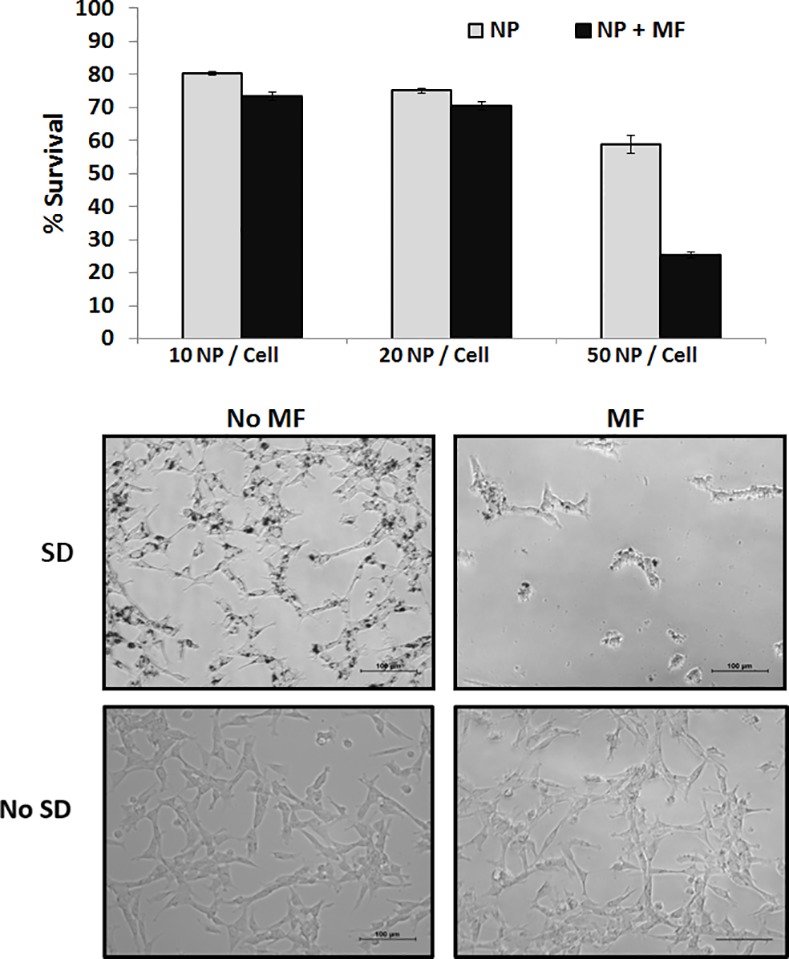
A: MTT assay of HB1.F3.CD cells. 10, 20, and 50 SD particles per cell were added into HB1.F3.CD cells for 24 hours. MF treatment consists of applying a 1T field rotating at 20 Hz for 30 min. B: Representative optical microscopy images of HB1.F3.CD cells before and after MF treatment. The top panel contains SD and the bottom panels are control without SD.

In order to investigate the potential application of SD for cancer treatment, we designed a combination system using NSCs loaded with magnetic particles (SD-NSCs) for controlled delivery and targeted glioma treatment. A schematic of the SD loading can be found in Fig 1. Research grade HB1.F3.CD NSCs, (used to generate a GMP grade NSC bank used in clinical trials for patients with recurrent high-grade gliomas (ClinicalTrials.gov Identifier: NCT02015819). The particles were characterized using SEM of the SD alone and TEM of the SD within the cells (S1 Fig). To determine the particle loading and toxicity within NSC cells, we dosed the cells with 10 SD/cell, 20 SD/cell or 50 SD/cell and exposed them to a rotating MF trigger. As shown in Fig 2A, the MTT analysis of the cells incubated for 24h revealed that the SD are able to load into the NCSs and result in survival rates of 80% and 75% with 10 SD/cell and 20 SD/cell without MF trigger. The NSCs loaded with 50 SD/cell exhibited 58% survival without MF trigger and only 25% after MF exposure (30min, 1Tesla). From the MTT assay we were able to determine that 50 SD/cell resulted in the greatest cell death with higher than 50% viability most likely due to excess loading. Further optimization is needed to increase NSC viability so that more cells can potentially be delivered. This dose was used in all subsequent experiments, however, actual internalization of the SD through Image J analysis from over 500 cells was determined to be approximately 29.76 SD/cell, an example of the image used can be found in S2C Fig. We used a loading ratio of 50 SD/cell for subsequent experiments, as once the NSCs traveled to the site of interest, there were enough SD particles delivered and released for effective uptake into the glioma cells. The 10 SD/cell and 20 SD/cell did not show a significantly lower survival rate after MF treatment, which is likely due to the number of particles in the cells not being sufficient to cause membrane disruption during MF treatment.

In Fig 2B, we used differential interference contrast microscopy to visualize the effects of the cells with and without SD after 30 min MF treatment. As shown in the representative image, the cells are attached to the dish with the SD localized internally, and after treatment the majority of the cells have died and thus detached due to the motion of the particles which compromised the membrane. The MF testing station has been constructed to produce a uniform 1 Tesla MF diametrically across the air gap, and thus the cell detachment is solely due to the ability of the particles to compromise the cell membrane resulting in cellular death and not due to a strong magnetic force pulling the particles out of the cells.

### Localized MF trigger causes cell membrane permeability of NSC

**Fig 3:**
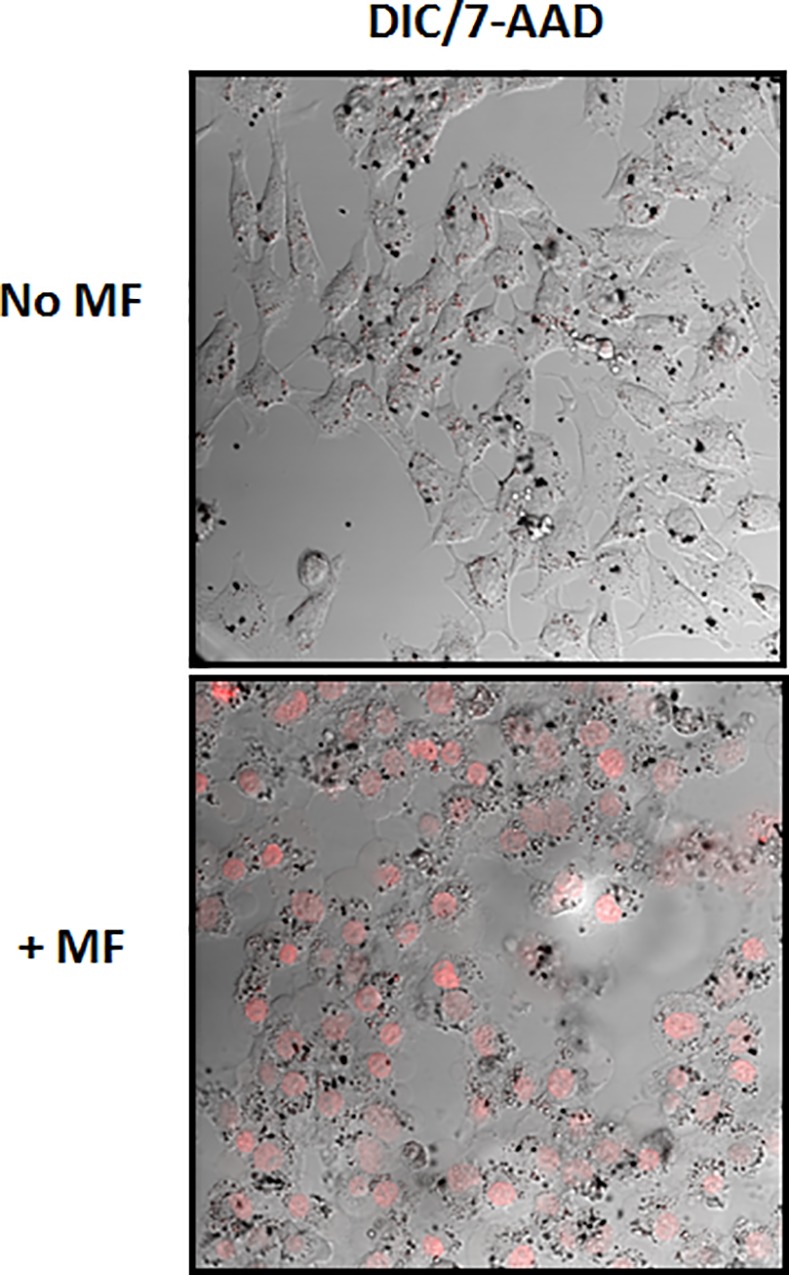
7-AAD staining of neural stem cells. The cells are incubated with 50 SD/cell for 24h and then receive or not the MF treatment for 30sec. The SD alone do not cause damage to the cells, only when exposed to a MF.

In order to understand the release of the SD from the NSCs and to ensure that the SD were released from dying cells rather than undergoing exocytosis from healthy cells, we looked at the health of the cells 24h after MF treatment. Under rotating MF, the particles rotate to cause membrane damage to the cell surface. To determine whether the membrane of SD-loaded NSCs is disrupted during MF treatment, we stained the cells under confocal microscopy conditions with 7-AAD. As shown in Fig 3, the SD alone (SD + no MF) do not contribute to compromised membranes, which suggest that the cells are compromised only during the MF treatment.

**Fig 4:**
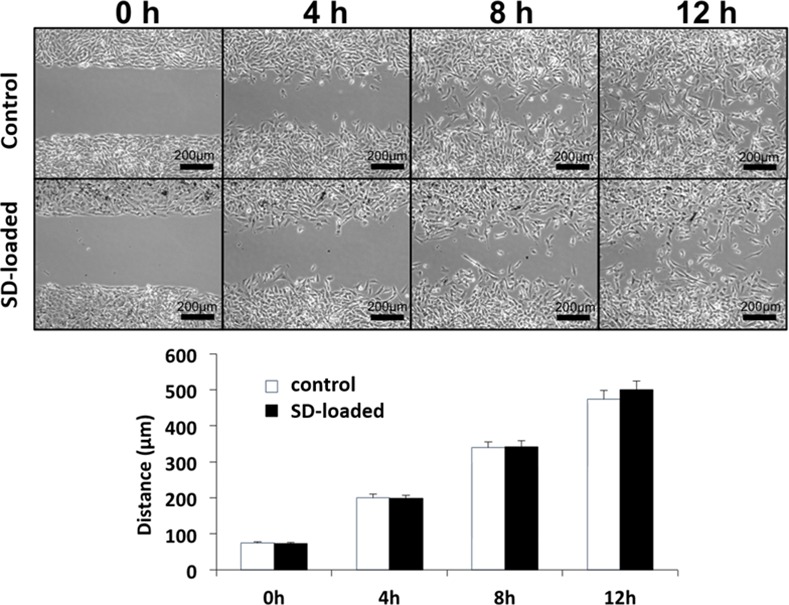
SD do not impair NSC migratory function. Cells are plated in a migration chamber for 24h before removal of the barrier. The control NSC and the NSC loaded with 50SD/cell demonstrate no difference in migration ability.

With the mechanism for field-induced NSC destruction established, we needed to verify that the presence of the SD particles at 50 SD/cell does not impact NSC migratory function. To understand whether loading with magnetic particles affects NSC tumor-tropism, migration assays were carried out in triplicate with 10,000 cells plated within each barrier chamber and allowed to adhere. After 24h the barrier was removed and NSC migration into the blank space was studied for 12h. As demonstrated in Fig 4, SD loaded and control NSCs did not show any significant difference in the distance migrated over time. After 12h, both the control cells and the SD-loaded cells were found at an average distance of 400μm past the reference point (the solid cell fronts easily distinguished at 0h), indicating that the SD within the cells did not inhibit the migration function of the NSC. High magnification of the particles in the cells at 4h, 8h, and 12h can be found in S3 Fig and the negative contrast image in S4 Fig.

### NSCs are able to efficiently release SD resulting in uptake with glioma cells

**Fig 5:**
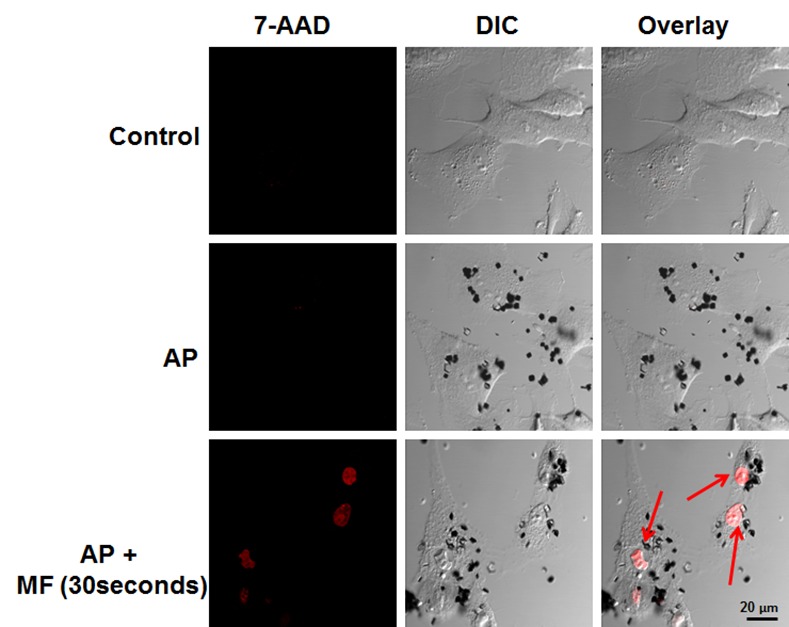
SD uptake and MF effect on glioma cells, U87. The cells are treated with 50SD/cell and allowed to incubate for 24h. Afterwards, the cells are exposed to MF treatment for 30 seconds and then stained with 7-AAD. The cells that receive SD + no MF do not have compromised membranes. The cells with SD and MF stain red.

**Fig 6:**
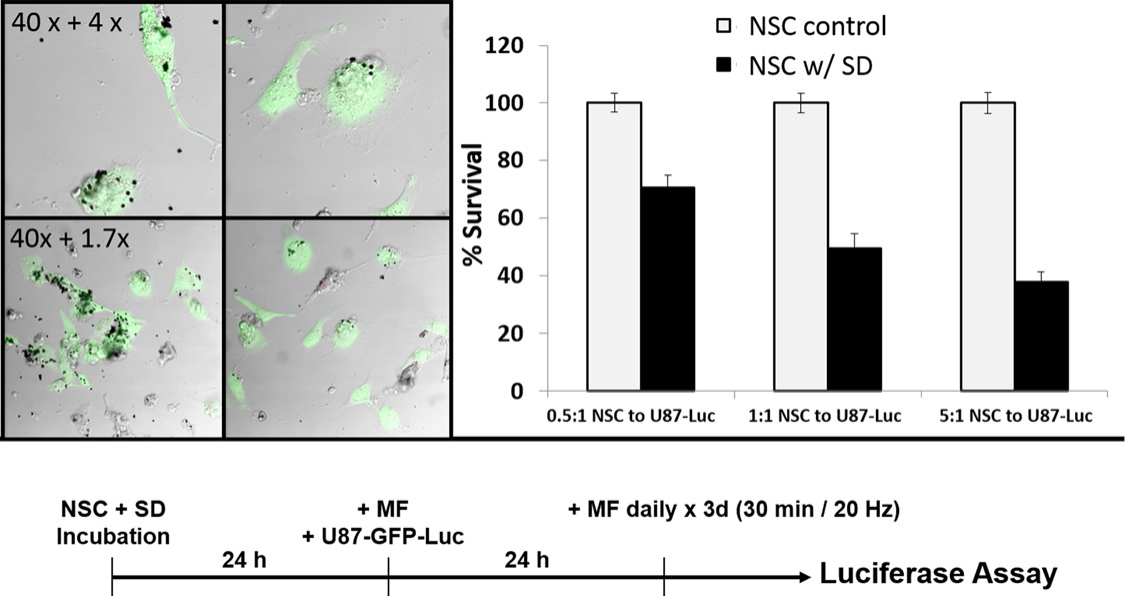
A: Confocal microscopy images of U87 glioma cells uptake of released SD particles (50 SD/NSC). The U87 cells (green) are able to effectively internalize particles that have been released by the NSCs. NSCs (grey) are visibly damaged. B: U87 survival rate determined by luciferase. C: Timeline of the experiment.

To determine whether glioma cells are able to take up the SD and undergo the same field-mediated destruction as the NSCs, we tested the U87 glioma cells with the particles in the presence of a MF. As shown through 7-AAD staining in Fig 5, the membranes of the U87 glioma cells incubated with SD are compromised after 30s of MF treatment, additional images can be found S5 Fig. As SD compromise the cell membrane, we observed that the cells do not immediately release the SD. The NSCs require 24h before 25% viability is observed. The control U87 cells without SD that have undergone the MF treatment (no SD + MF) as well as the cells with SD that have had no MF treatment (SD + no MF) had no cell death. This demonstrates that the particles under MF treatment are the cause of damage to the glioma cells. We next determined whether the SD can be redistributed to glioma cells after NSC cell death. 10,000 NSCs were mixed with SD at a concentration of 50 SD/cell and incubated for 24h. The NSCs were then exposed to MF treatment for 30 min in order to release the SD, and the then immediately plated with U87-GFP-Luc cells and incubated for 24h to allow uptake of the released SD. Fig 6A shows an optical image of SD released by NSCs being successfully taken up by U87 cells (additional images can be found Fig 6. The resulting U87 cells received a 30 min MF treatment daily for 3 days and the resulting luciferase was measured to determine the amount of viable glioma cells at the end of treatment. See Fig 6C for a schematic timeline of the experiment. As shown in Fig 6B, NSCs without SD (NSC + no SD + MF) had no toxic effect on the glioma cells alone. Furthermore, the SD loaded NSCs (NSC + SD + MF), demonstrate a significantly lower survival rate upon MF treatment; the SD loaded NSCs (10,000 cells) result in 70%, 49%, and 37% viability of the U87 cells when plated at a ratio of 0.5:1, 1:1, and 5:1 (20,000, 10,000 and 2,000) NSC to U87 cells respectively, (p < 0.05). In addition, NSCs loaded at 50 SD/cell were enough to kill half of the population of glioma cells. These results indicate that SD are able to efficiently be taken up by glioma cells following triggered release from NSCs, despite any potential presence of protein coronas or other cellular components that have electrostatically bound to the surface of the SD. This important result indicates that glioma cells readily take up SD which are able to induce cellular damage after MF treatment.

## Conclusion

In summary, we successfully demonstrate a magnetically controlled and targeted delivery strategy via SD-loaded NSCs. The delivery of particles across the blood brain barrier has proven difficult and furthermore, particle therapies are often limited by the number of therapeutic particles that can be delivered. NSCs are able to migrate across the blood brain barrier and have recently become viable candidates for drug delivery utilizing their natural tumor tropism. The SD are unique because they do not need to be functionalized with any therapeutic that can potentially degrade, and are able to be re-internalized into tumor cells for repeated delivery and treatment. In addition, using an externally applied MF that is able to successfully trigger release of the particles from the NSC has great potential for future drug delivery therapies. The particles alone are not toxic to the cells and have no disruptive abilities as long as no MF is applied. These sets of experiments demonstrate the potential for the combination NSC + SD therapy by utilizing the natural ability of the NSCs to target tumor sites as well as effectively deliver SD for glioma destruction.

## Supporting Information

S1 Fig SEM of free spinning disks. (TIF)

S2 Fig Characterization of the spinning disks. (TIF)

S3 Fig Time images of the migration of cells over time. (TIF)

S4 Fig Images of the cells and the negative contrast image. (TIF)

S5 Fig Additional 7-AAD staining of NSC. (TIF)

S6 Fig Additional images of confocal microscopy images of U87 glioma cells uptake of released MD particles. (TIF)
