# Supplementary figures and images for: Controlled Payload Release by Magnetic Field Triggered Neural Stem Cell Destruction for Malignant Glioma Treatment
Source: PLoS One. 2016 Jan 6;11(1):e0145129. doi: 10.1371/journal.pone.0145129 (PMC4703386; doi:10.1371/journal.pone.0145129)

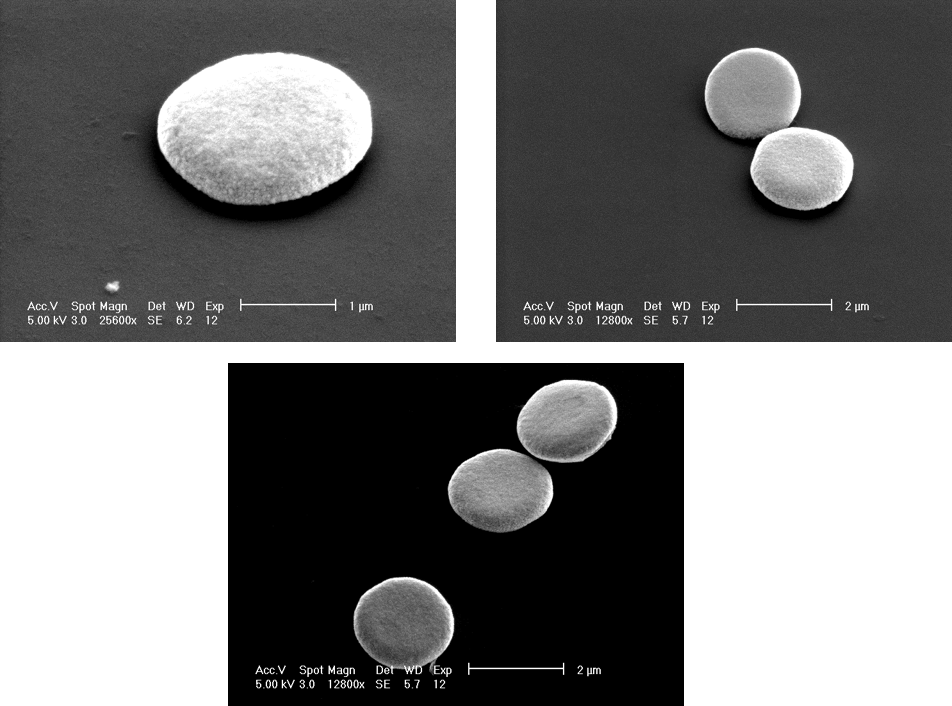

Supplement: S1 Fig — (TIF) [file pone.0145129.s001.tif]

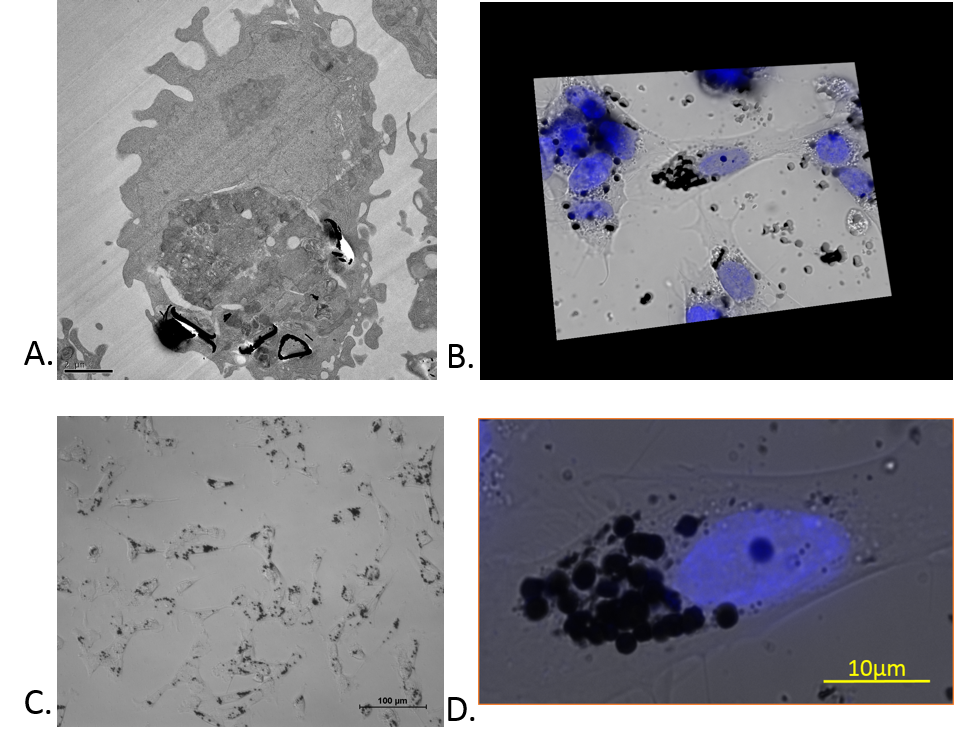

Supplement: S2 Fig — (TIF) [file pone.0145129.s002.tif]

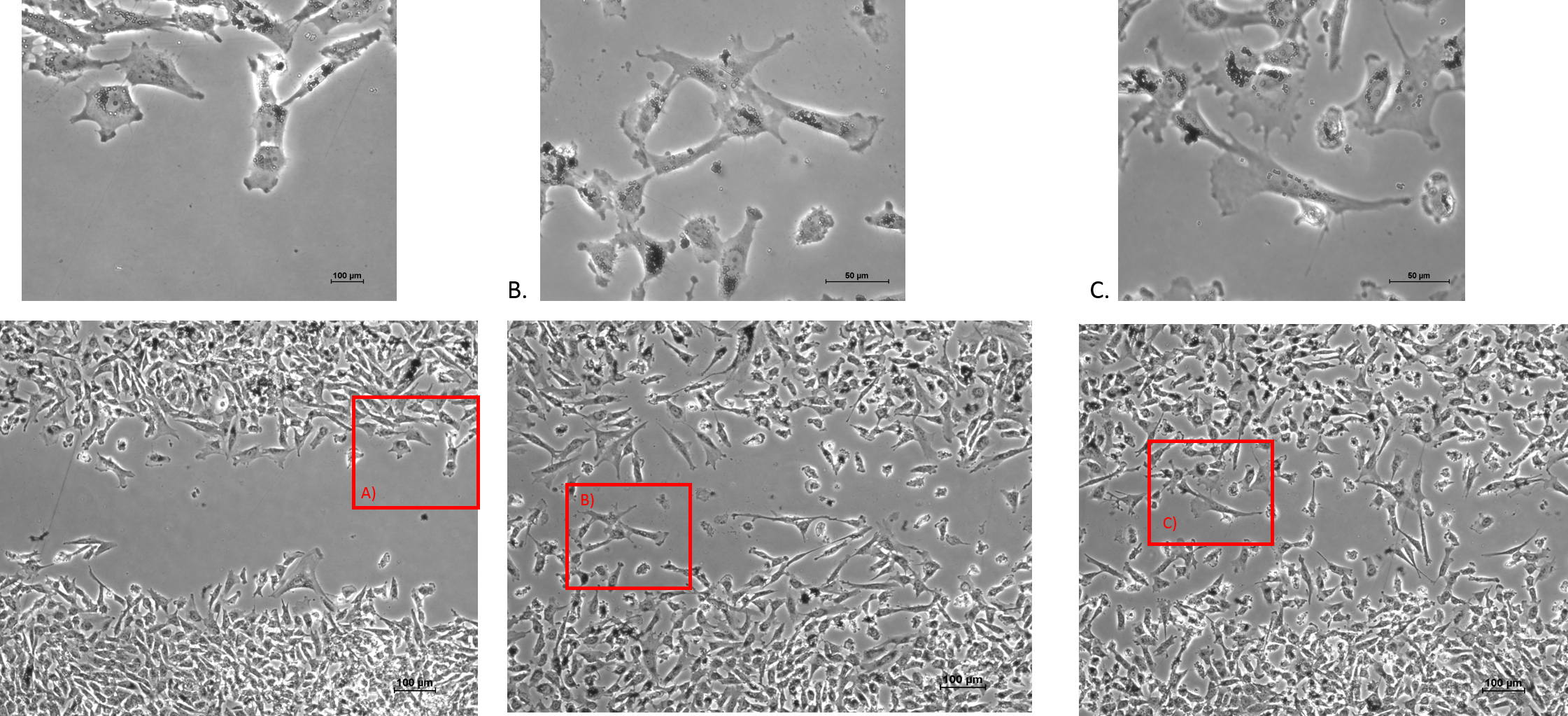

Supplement: S3 Fig — (TIF) [file pone.0145129.s003.tif]

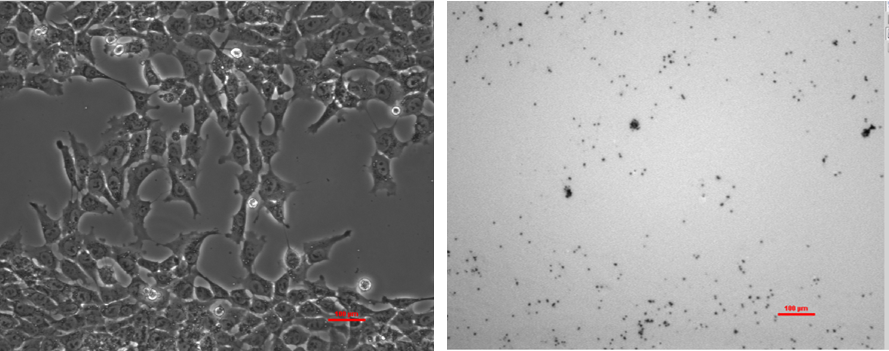

Supplement: S4 Fig — (TIF) [file pone.0145129.s004.tif]

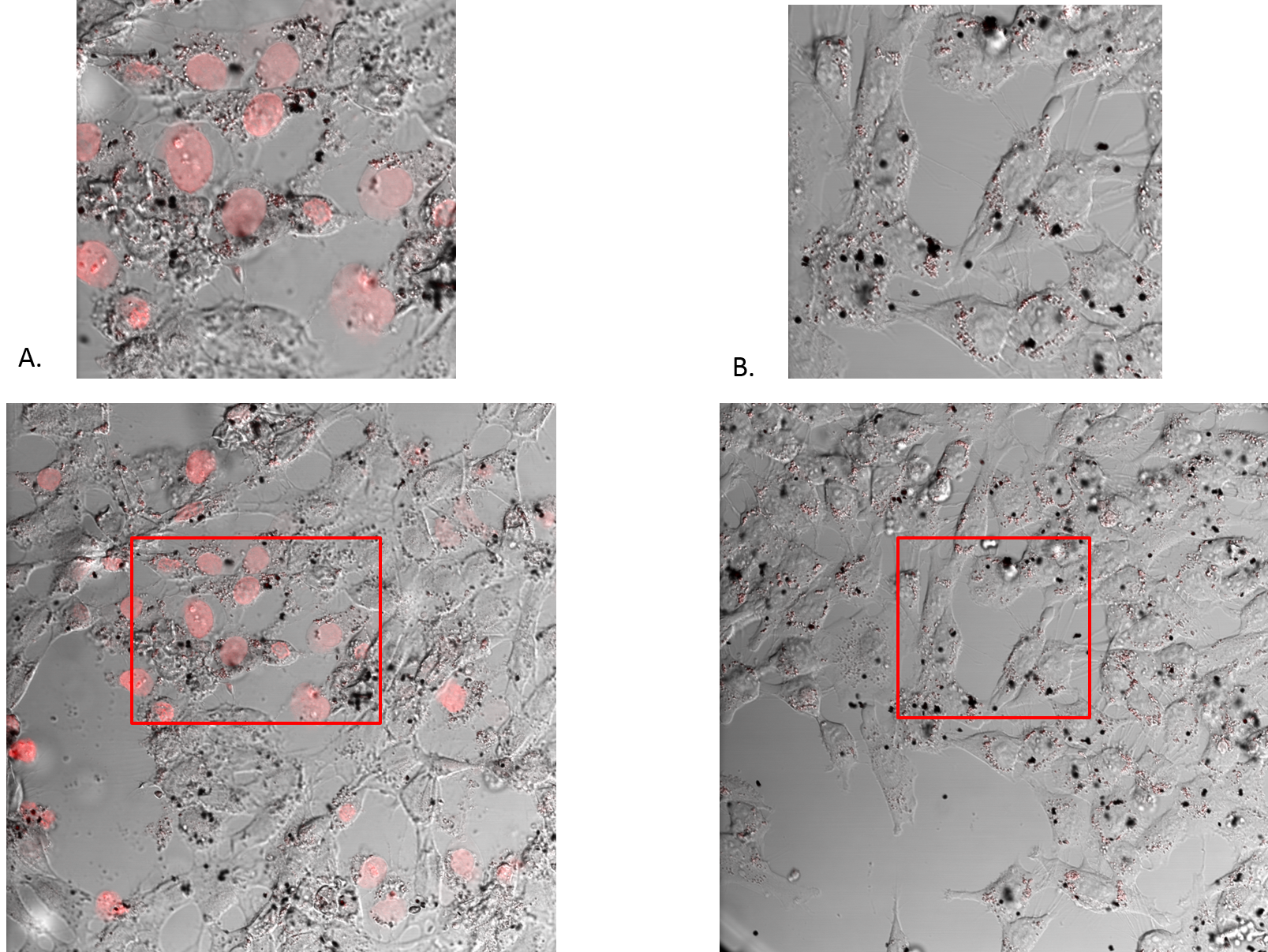

Supplement: S5 Fig — (TIF) [file pone.0145129.s005.tif]

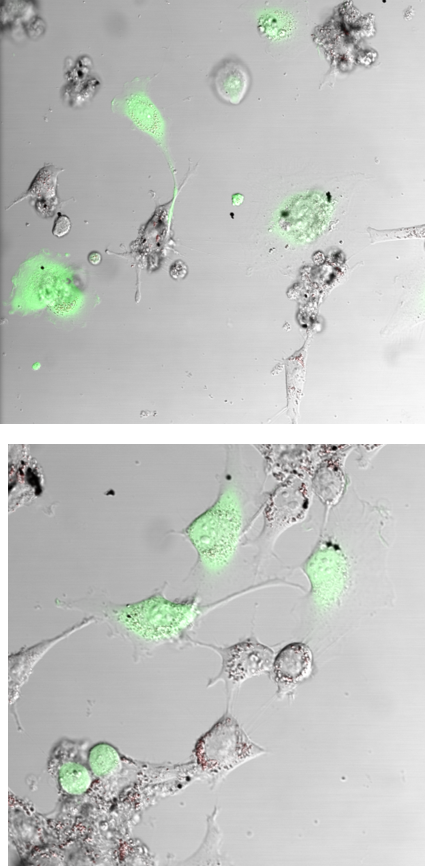

Supplement: S6 Fig — (TIF) [file pone.0145129.s006.tif]
